# Exchange Bias Optimization by Controlled Oxidation of Cobalt Nanoparticle Films Prepared by Sputter Gas Aggregation

**DOI:** 10.3390/nano7030061

**Published:** 2017-03-11

**Authors:** Ricardo López Antón, Juan A. González, Juan P. Andrés, Peter S. Normile, Jesús Canales-Vázquez, Pablo Muñiz, José M. Riveiro, José A. De Toro

**Affiliations:** 1 Instituto Regional de Investigación Científica Aplicada (IRICA) and Departamento de Física Aplicada, Universidad de Castilla-La Mancha, 13071 Ciudad Real, Spain; j.a.gonzalez@uclm.es (J.A.G.); juanpedro.andres@uclm.es (J.P.A.); peter.normile@uclm.es (P.S.N.); pablo.muniz@uclm.es (P.M.); riveiro.jm@hotmail.com (J.M.R.); 2 Instituto de Energías Renovables, Universidad de Castilla-La Mancha, 02071 Albacete, Spain; jesus.canales@uclm.es

**Keywords:** magnetic nanoparticles, core-shell nanoparticles, Co/CoO, exchange bias, magnetic properties

## Abstract

Porous films of cobalt nanoparticles have been obtained by sputter gas aggregation and controllably oxidized by air annealing at 100 °C for progressively longer times (up to more than 1400 h). The magnetic properties of the samples were monitored during the process, with a focus on the exchange bias field. Air annealing proves to be a convenient way to control the Co/CoO ratio in the samples, allowing the optimization of the exchange bias field to a value above 6 kOe at 5 K. The occurrence of the maximum in the exchange bias field is understood in terms of the density of CoO uncompensated spins and their degree of pinning, with the former reducing and the latter increasing upon the growth of a progressively thicker CoO shell. Vertical shifts exhibited in the magnetization loops are found to correlate qualitatively with the peak in the exchange bias field, while an increase in vertical shift observed for longer oxidation times may be explained by a growing fraction of almost completely oxidized particles. The presence of a hummingbird-like form in magnetization loops can be understood in terms of a combination of hard (biased) and soft (unbiased) components; however, the precise origin of the soft phase is as yet unresolved.

## 1. Introduction

Since its discovery by Meiklejohn and Bean in 1956 in partially oxidized Co particles [1], exchange bias (EB) has been observed in a variety of nanostructured systems. It occurs when two materials with significantly different magnetic anisotropy, usually one being ferromagnetic (FM) and the other antiferromagnetic (AFM), are exchange-coupled at their interface [2,3]. Such exchange coupling manifests itself through different phenomena, typically including shifts of the hysteresis loop along the applied magnetic field axis, *H_E_*, and the magnetization axis (vertical shift, VS), as well as a coercivity enhancement. EB is extensively exploited in spintronic devices such as spin valves [2,4], and holds great potential for, e.g., novel nanostructured permanent magnets [5]. The effect has been therefore intensively studied, mainly in the case of thin films/multilayers [4], including discontinuous multilayers [6], but also in nanoparticles produced by a variety of techniques [7,8,9,10,11], where exchange bias has been proposed as a strategy for the stabilization of the FM core with a view, e.g., to delaying the superparamagnetic limit on magnetic recording [7,12]. In particular, Co/CoO nanoparticles (NPs) have been fairly intensively studied, as the Curie and Néel temperatures are convenient for magnetic characterization with standard equipment. Cobalt NPs can be either fully oxidized to cobalt oxides (CoO or Co_3_O_4_) or partially oxidized forming (i) core-shell (metallic Co cores surrounded by a shell of native oxide(s)) [8,9], (ii) hollow [8,10] or (iii) yolk [8] structures depending on the particle size, the annealing temperature and the abundance of oxidants. Additionally, NPs with Co and CoO phase mixing and high exchange bias fields can be produced by reactive gas-phase aggregation [11]. 

Both the thickness of the CoO shell and the diameter of the Co core are crucial to maximize the H_E_ value. Obviously, an optimum core/shell geometry must exist as a function of oxidation between the pure Co and CoO extremes (both with zero bias) [13], but its occurrence at a specific Co/CoO ratio is related to complex details of the interface spin structure, in turn often determined by the size of the CoO shell. Two models to explain the variation of H_E_ with the size of the FM core and the AFM shell are those due to Dobrynin et al. [14,15], who found a critical size below which EB vanishes (based on energy considerations), and to Feygenson et al. [16], whose analysis suggests that lattice strain induces a net (uncompensated) moment of the (100) planes of the oxide at the core-shell interface (achieved via the strain dependence of the relative amplitudes of two different antiferromagnetic modulations in the shell, leading to spin canting). It is the variation of that moment with the shell thickness that, according to those latter authors, plays a decisive role in the production of a maximum in the EB field. Their reported EB maximum (with *H_E_* ≈ 7 kOe) from the variation of the Co/CoO ratio (achieved by flowing different amounts of oxygen through the NP liquid solution) is a scarce example of such EB optimization within the literature. However, only four non-zero *H_E_* data points were presented, the same number as in the study by Kovylina et al. [13], who also reported a maximum in the EB field (obtained, this time, across a five-order-of-magnitude variation in the oxygen pressure during the laser-ablation deposition of the NPs), albeit with a smaller value (*H_E_* ≈ 900 Oe). In the present work we have prepared a porous film of Co/CoO magnetic NPs (5–6 nm in diameter) by gas-phase aggregation, and have monitored the evolution of the magnetic properties upon the progressive oxidation of the film by annealing at 100 °C in air during progressively longer times, a simpler method than those previously mentioned, but nevertheless allowing a remarkable fine-tuning of the Co/CoO volume ratio, which leads to a sharp peak in the evolution of *H_E_* with the annealing time (and therefore with the degree of oxidation).

Additionally, we observe the appearance of a soft FM phase in the field-cooled hysteresis loops, leading to the so-called “hummingbird-like” shape [8]. We critically review previously suggested explanations for the origin of this phase before ruling out such interpretations in the case of our present data.

## 2. Results and Discussion

**Figure 1:**
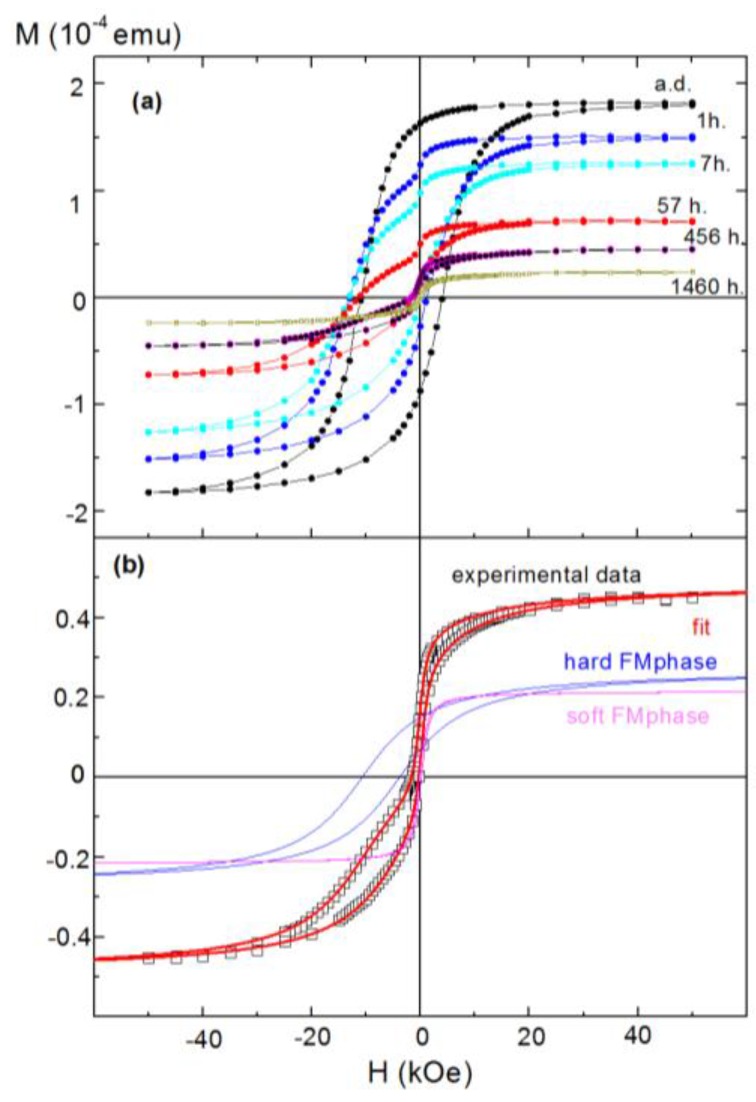
(**a**) Hysteresis loops, recorded at 10 K after cooling from room temperature under an applied field of 50 kOe, measured in the as-deposited sample (ad) and after air-annealing at 100 °C for different (cumulative) times; (**b**) Example of one of the fits to the hummingbird-like loops (that of the 456 h annealed sample), obtained using a combination of a hard and a soft FM phase (after correcting the data for vertical shift).

Figure 1a displays FC hysteresis loops measured at 10 K after selected annealing times. The loop corresponding to the as-deposited film (measured minutes after removing the film from the vacuum chamber) already shows a large *H_E_*, indicating partial oxidation of the samples, as expected from the high reactivity of porous films of metallic nanoparticles. A low-field step in the upper branch hysteresis loop appears after annealing for 1 h, giving the loop a characteristic “hummingbird-like” shape (we shall discuss this feature in more detail below). We have successfully fitted all hysteresis loops with two FM contributions, namely a hard (and strongly biased) FM phase and a soft unbiased component, using the following empirical expressions [17]:

(1) $$M_{L\left(R\right)}=M_{H,L\left(R\right)}+M_{S,L\left(R\right)}$$

(2) $$M_{H,L\left(R\right)}=\frac{2M_{S,H}}{\pi }\arctan\left\{\frac{H+H_{E,H}\pm H_{C,H}}{H_{C,H}}\tan\frac{\pi S_{H}}{2}\right\}$$

(3) $$M_{S,L\left(R\right)}=\frac{2M_{S,S}}{\pi }\arctan\left\{\frac{H\pm H_{C,S}}{H_{C,S}}\tan\frac{\pi S_{S}}{2}\right\}$$

where the subscripts *H* and *S* refer to hard and soft contributions and *L*(*R*) indicates the left (right) branch, *M_S,H_* and *M_S,S_* are the saturation moments, *H_E,H_* is the EB field (hard component only), *H_C,H_* and *H_C,S_* are the coercivity field (both components), and *S_H_* and *S_S_* are the squareness parameters. The ± is positive for an *L* branch and negative for an *R* branch. Both branches of the loops were fitted simultaneously after correcting for vertical shift. As a representative example, Figure 1b plots the two contributions resulting from such fitting for an annealing time *t_A_* = 456 h. In all cases the soft FM phase presents a very low *H_C,S_* (below 100 Oe), whereas the hard FM phase exhibits high coercivity and exchange bias. 

**Figure 2:**
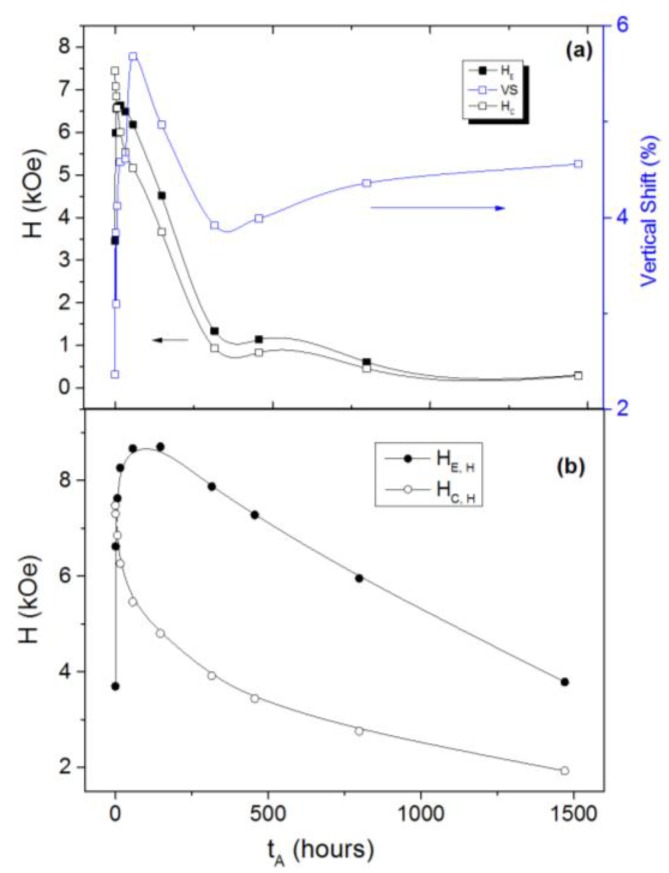
Annealing time dependence of the exchange bias and coercive fields, extracted from both the as-measured loops (upper panel, Figure 2a) and of the hard component extracted from the fits as exemplified in Figure 1b (lower panel, Figure 2b), as well as of the vertical shift (blue squares). The lines are guides for the eye.

Figure 2 shows the evolution of the EB and coercive fields with the annealing time, with both values extracted directly from the raw loops (*H_E_* and *H_C_*; lower panel, Figure 2b) and those obtained from the fitted hard component (*H_E,H_* and *H_C,H_*; lower panel, Figure 2a). Also plotted in Figure 2a are the VS values. Each method of extracting the EB field clearly results in a single well-defined peak, although the peak value, position and shape differ between the two methods: *H_E,H_* takes a maximum value of around 8.5 kOe at 80 h of annealing, while the raw loop value *H_E_* shows both a lower peak value (6.5 kOe) and a lower position (17 h).Therefore, qualitatively speaking, our observation of a peak is not an artifact related to the presence of the soft phase. Rather, the maximum in the EB field results from an optimum Co/CoO volume ratio and the concomitantly favorable interface conditions, as discussed next in view of the two aforementioned models.

Dobrynin et al.’s [14,15] model considers the different weights of several energy terms (Zeeman, anisotropies of the AFM and FM phases, and FM-AFM exchange at the interface) to determine the size range where EB exists, obtaining a critical size of the core below which EB vanishes. This simple model could roughly explain the trend found in our results. However, the model relates all vertical shift values to very small Co cores completely pinned by the CoO AFM phase (not reversing even at large applied fields), neglecting the contribution of pinned uncompensated spins [18]. In our samples we find non-vanishing VS values over the entire range of annealing times (see Figure 2), with a maximum at a position (annealing time) close to that of either peak in the EB field (*H_E_* or *H_E,H_*). Small and completely pinned Co cores could account for the increase in VS at longer annealing times (Figure 2b); however, it is unlikely that they account for the evolution of this parameter over the entire time range. 

The model provided by Feygenson et al. [16], namely of shell thickness–dependent lattice strain–induced uncompensated (100) oxide planes at the core-shell interface as a mechanism to explain the occurrence of a maximum in the EB field upon varying shell thickness, is appealing in the context of our present results since it offers the following explanations. In under-oxidized films (i.e., at low annealing times), the very thin CoO shells are expected to possess an AFM structure considerably distorted from that of bulk CoO, and hence to possess significantly lower anisotropy, implying that the uncompensated moments at the core-shell interface will tend to follow the core upon applied field reversal, which may explain the initially low EB field in Figure 2. As the shell thickness increases with the annealing time, strain-induced uncompensated moments are still expected but will now be impeded from free rotation with the core by their exchange coupling with the thicker (more anisotropic) AFM shell, explaining the increase in the EB field towards a maximum in Figure 2. Further increase of the shell thickness will eventually reduce the amount of uncompensated spins at the interface, as the AFM structure approaches that of bulk CoO with fully compensated (100) planes, explaining the subsequent decrease in the EB field in Figure 2.

It should be mentioned that Feygenson et al. [16] were able to affirm the realization of a near-epitaxial core-shell interface parallel to the (100) planes of CoO, resulting from the “relatively low” oxidation temperature employed (180 °C) together with the fact that their Co particles (prepared by a chemical method) were oxidized in suspension. Given that the oxidation temperature used in our present study is also relatively low (100 °C), it is possible that a similar near-epitaxial growth takes place. However, the thickness of the resulting CoO shells in our system is likely to be far less uniform than in the core-shell material prepared by Feygenson et al., due to the oxidation of porous NP films, in other words, due to the NP agglomeration effects inherent in our use of a physical synthesis method (sputter gas aggregation) without the co-deposition of a matrix material to separate the Co NPs (such agglomeration is greatly inhibited in Feygenson et al.’s study by the attachment of surfactant shells, afforded by their use of a chemical synthesis method). It is therefore reasonable that, while the evolution of the EB field with the annealing time can be qualitatively understood from the insights provided by Feygenson et al., certain aspects of the magnetization loops obtained in the present study differ from those in Feygenson et al.’s study. At least two such differences are identified: no vertical shifts were found in Feygenson et al.’s study (while, as mentioned, we found non-vanishing VS across our entire range of oxidation times), and hummingbird-like loops were also absent (while they were prevalent in the present study). 

Two types of uncompensated spins may be expected over the range of oxidation times in the present study: *pinned spins*, unaffected by the applied magnetic field, giving rise to a significant EB field and also to the VS, and *unpinned* or *rotatable spins*, which are dragged by the FM and are thus related to the enhancement of the *H_C_* [19,20]. The monotonic decrease of coercivity (*H_C_* or *H_C,H_*) with the annealing time may therefore be (at least partially) related to a reduction of rotatable uncompensated spins at the core-shell interface as the thickness of the shell increased and the AFM structure of bulk CoO was approached. Regarding the VS, we found a more complex behavior, with an increase up to the EB peak (which appeared a trivial result considering that VS results from the same pinned uncompensated spins producing the EB field), followed by a decrease and then a final increase for the longest annealing times. The decrease would still follow the behavior of *H_E_* (*t_A_*), now reflecting a decreasing number of (both pinned and unpinned) uncompensated spins, but we need to resort to Dobrynin’s model [14] in order to explain the final increase in VS, where almost fully oxidized particles would present very small Co cores strongly pinned by the CoO AFM phase (i.e., with a coupling energy larger than the Zeeman term at the maximum applied field).

Moving now to the origin of the hummingbird-like loops and the possible interpretation in terms of a soft phase, we should mention that in several previous studies, similarly asymmetric hysteresis loops were *not* explained in terms of a soft FM phase. In the Co/CoO porous NP films studied by Dobrynin et al., step features in magnetization loops were found at larger applied field values than in the present study, a behavior attributed to the existence of two hard FM phases instead of a hard and a soft phase, with a very relevant role of the AFM matrix aligning FM clusters and biasing the loops of clusters in opposite directions with oppositely oriented magnetizations. Chandra et al. [9], on the other hand, related such asymmetry in the loops (again of Co/CoO NPs) observed below a temperature corresponding to the freezing of shell moments along the cooling field direction, introducing an additional anisotropy energy that dominated over the Zeeman energy in the returning branch. Sun et al. [21] observed a similar behavior in FeO/Fe_3_O_4_ NPs, and linked it to a different magnetic switching process in the two branches of the loop: domain wall nucleation in the descending branch and magnetic rotation in the ascending branch (although this explanation has been mainly used for explaining other kinds of loop asymmetry, e.g., that in Reference [22]). Ong et al. explained their results in Fe/Fe_3_O_4_ NPs [23,24] with a reduction of the Zeeman energy of the ferrimagnetic Fe_3_O_4_ domains in the shell at low fields.

**Figure 3:**
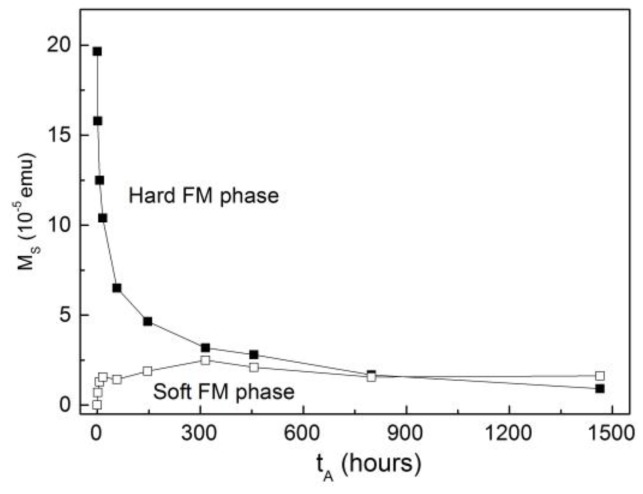
Annealing time dependence of the saturation moment of the hard and soft components of the hysteresis loop.

However, in our case the existence of a soft FM phase seems fairly clear, as evidenced by the ability to systematically decompose loops (as in Figure 1b) over the progressive oxidation of the NP film. In particular, Figure 3 displays the annealing time evolution of the saturation moments of both the hard and soft components of the measured loops. As expected, *M_S,H_* decreased strongly upon oxidation with longer annealing times, and therefore could be ascribed to the down-scaling of the Co cores upon oxidation. In contrast, *M_S,S_* increased significantly only for the first few hours of annealing, after which it remained fairly constant. Thus, as it was uncorrelated with the volume of either the Co core or the CoO shell, this soft phase appeared to be related to the early formation of the CoO shells.

**Figure 4:**
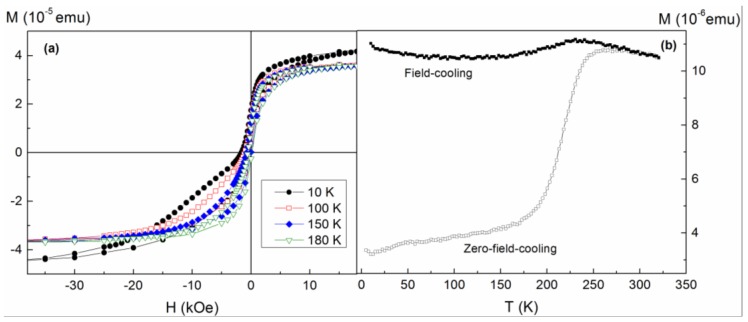
(**a**) Hysteresis loops of the sample annealed for 456 h recorded at different temperatures after field-cooling from 300 K (only the data over a limited H range are presented) and (**b**) zero-field-cooled and field-cooled magnetization curves measured in the same sample in an applied field of 500 Oe.

The precise origin of the soft FM phase cannot, at this stage, be clearly established. Simeonidis et al. linked it to the presence of a Co_3_O_4_ phase in their Co/CoO samples, evidenced by the disappearance of the phenomenon for temperatures above the Néel temperature of Co_3_O_4_ (around 50 K) [8]. However, in hysteresis loops recorded at different temperatures for the sample annealed for *t_A_* = 456 h (see Figure 4a), both phases were observed up to EB onset temperature (*T_B_* (*H_E_*) ≈ 180 K). Hence, in our case the soft phase does not correspond to Co_3_O_4_. The explanation suggested by Tracy et al. [25], of superparamagnetic (SPM) clusters at the interface uncoupled to both the FM core and AFM shell, also appears irrelevant to our present system, since the loops plotted in Figure 4a present a low-field susceptibility of the soft phase that does not decrease significantly with temperature (as would be expected for SPM particles). Moreover, the magnitude of such susceptibility leads to cluster sizes unrealistically large given the small overall size of the nanoparticles. Another possibility that may be discarded is that given the size distribution of the NPs, the smallest NPs oxidize completely, supporting a small particle moment (due to surface uncompensated spins) with a magnetic response very different from that of the partially oxidized particles [26], since this scenario would lead to a monotonic increase of the saturation moment of the soft phase upon annealing (as an increasing fraction of the NPs became fully oxidized), irreconcilable with the observation of a fairly constant value achieved after only a few hours of thermal treatment (see Figure 3). Furthermore, the double-loop character of the hysteresis is not reflected as a double-feature in zero-field-cooled (ZFC) curves (see Figure 4b), where instead a single blocking event is observed very close to the EB onset temperature, indicating that the stability of the FM core is governed by its exchange coupling to the AFM shell.

## 3. Materials and Methods

**Figure 5:**
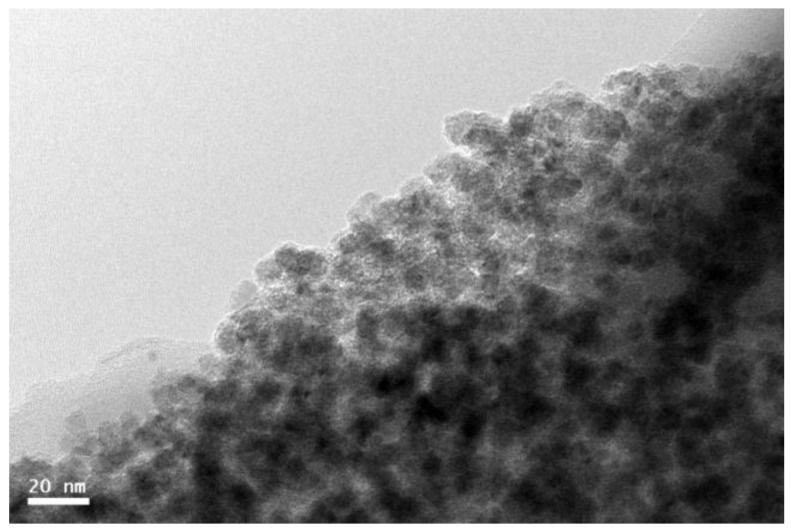
Transmission electron microscopy (TEM) micrograph of the nanoparticle film after annealing for 6 h.

Cobalt nanoparticles (NPs) were grown by sputter gas-phase aggregation on Si substrates, forming porous films without any protective capping. The cluster source (DC magnetron) was operated at 30 W with a flow rate of 40 sccm of the sputtering gas (Ar). The base pressure of the deposition chamber was 3 × 10^−7^ mbar, and the process pressure 1.3 × 10^−3^ mbar. The sample was grown during 16 min with a deposition rate (measured at the beam axis position using a quartz crystal monitor) of 1.4 Å/s, thus leading to a film thickness greater (considering porosity) than 140 nm. The mean particle size (including the CoO shell) is about 6 nm (see Figure 1), similar to Co particles produced under the same synthesis conditions in previous studies [27]. The same sample was annealed in air at 100 °C for progressively longer times (up to a total cumulative time of *t_A_* = 1464 h) in order to monitor the change of magnetic properties resulting from the controlled variation of the Co/CoO volume ratio. Figure 5 shows a Transmission electron microscopy (TEM) micrograph of an edge of a fraction of the film removed from the substrate after a 6 h annealing, illustrating the high degree of porosity of the sample.

Magnetic hysteresis loops were measured at 10 K up to a maximum applied field of 50 kOe using an EverCool MPMS-XL SQUID magnetometer (Quantum Design). These loops were recorded after field-cooling from room temperature in a saturating field (50 kOe) in order to measure the exchange bias field. After selected annealing times, field-cooled (FC) and zero-field-cooled (ZFC) magnetization curves were registered (in an applied field of 500 Oe upon heating from 5 to 300 K). The diamagnetic contribution from the silicon substrates and the vertical shift (shown separately) have been corrected in all the hysteresis loops shown below.

## 4. Conclusions

Porous films of Co/CoO nanoparticles were assembled by sputter gas-phase aggregation and subsequently annealed at 100 °C in air for progressively longer times. This has conveniently allowed the fine-tuning of the Co/CoO ratio in the samples and, hence, optimization of the exchange bias field, which exhibits a peak as a function of the annealing time (with a peak value of 8.5 kOe when removing a soft phase from the loops, and of 6.5 kOe otherwise). The evolution upon annealing of the EB field and the vertical shift can be qualitatively understood in terms of a combination of models previously reported by Dobrynin et al. [14,15] and Feygenson et al. [16]. A soft magnetic phase appearing after a short annealing time was thoroughly studied and several possible explanations for its occurrence have been ruled out. Its precise origin thus remains a mystery.
